# A distributed event-triggered framework for coordinating athlete autonomic states via wearable sensor networks

**DOI:** 10.3389/fpsyg.2026.1858564

**Published:** 2026-09-18

**Authors:** Chao Wang, Shixin Li

**Affiliations:** 1 Department of Physical Education, Dalian University of Science and Technology, Dalian, China; 2 School of Electrical Engineering, Dalian Jiaotong University, Dalian, China

**Keywords:** athlete autonomic monitoring, Bluetooth low energy, interpersonal autonomic physiology, multi-agent consensus control, physiological synchrony, wearable sensor networks

## Abstract

**Background:**

Interpersonal autonomic physiology (IAP) research has established that physiological synchrony (PS), the temporal interdependence of autonomic nervous system (ANS) activity across individuals, is an established phenomenon in team-based settings. However, PS in athlete cohorts has been studied exclusively as a passive observational variable; no framework actively regulates autonomic coherence under the communication constraints of wireless body area networks. This study integrates IAP and distributed control theory by formulating team-level autonomic coherence as a leaderless multi-agent consensus problem.

**Methods:**

Each wearable unit was modeled as a two-dimensional linear agent whose state encodes cardiovascular autonomic tone (parasympathetic index: HRV-RMSSD) and sympathetic arousal load (EDA skin-conductance level, SCL), consistent with the Banister Fitness-Fatigue Model. Initial model states were derived from ECG/EDA records of WESAD subjects S2-S7, used only to provide heterogeneous simulation initial conditions (correlations were directional, non-significant sanity checks: *r* = −0.71, *p* = 0.114 for RMSSD; *r* = 0.68, *p* = 0.137 for SCL; *N* = 6). Three engineering challenges were addressed: (i) BLE communication delays *h*_*c*_ ∈ [15, 150] ms; (ii) neuromuscular input delays *h*_*i*_ ∈ [150, 300] ms; and (iii) switching directed topologies from dynamic team formations. A distributed dynamic event-triggered mechanism (DETM) drove all agents toward consensus while transmitting data only upon detection of meaningful state deviation. Stability was established via a dual-integral Lyapunov-Krasovskii functional, with LMI feasibility verified by CVXPY (ε = 0.087). Simulations ran for 300 s under three delay scenarios with five initial-condition perturbation folds; battery life was estimated from a four-component nRF52840 energy model.

**Results:**

The proposed DETM reduced BLE transmissions by 38.38% in the nominal case while achieving convergence of the normalized model states within 4–7 s. A Zeno-free guarantee (*T*_min_ = 0.06 s) and a theoretical battery-life estimate of about 36 days were obtained. The ablation analysis attributed the largest communication saving to the dynamic internal variable ρ_*i*_(*t*) and the largest convergence-time improvement to the dual-delay structure. Five initial-condition perturbation folds indicated numerical robustness to the starting states.

**Discussion:**

These findings support control-theoretic and network-level feasibility only. Athlete studies are required before physiological benefits, safety, or real-world effectiveness can be inferred.

## 1 Introduction

The study of interpersonal autonomic physiology (IAP)—defined as the temporal relationship between the autonomic nervous system (ANS) activity of two or more people—has produced a robust body of evidence across the past six decades (Palumbo et al., 2017). From early observations of heart rate (HR) and electrodermal activity (EDA) correlated with therapist and patient during psychotherapy (Di Mascio et al., 1955; Coleman et al., 1956) to contemporary studies of HRV synchrony in military teams during operational tasks (Elkins et al., 2009) and respiration coherence in choral singers (Müller and Lindenberger, 2011; Vickhoff et al., 2013), the accumulated literature reveals a common principle: the ANS of individuals engaged in joint tasks is not physiologically independent. As Palumbo et al. (2017) concluded in their systematic review of 61 empirical studies, physiological synchrony (PS)—the interdependent or associated autonomic activity between individuals—appears to be a transient state that varies with context, relationship type, and task demands, and is informative of psychosocial constructs, including empathy, attention, cohesion, and team performance (Henning et al., 2001; Elkins et al., 2009; Walker et al., 2013).

In team sports, however, the application of IAP methods remains almost entirely observational. Practitioners and researchers monitor individual athlete physiology continuously via wearable ECG and EDA systems (Bourdon et al., 2017; Halson, 2014; Cardinale and Varley, 2017). However, the interpersonal dimension of these data is rarely exploited. The relevant IAP literature on teammates (summarized in Table 4 of Palumbo et al. 2017) shows that PS between team members is predictive of team performance (Henning et al., 2001; Henning and Korbelak, 2005), correlates with empathy and social presence (Järvelä et al., 2013), and varies with competitive vs. cooperative task framing (Chanel et al., 2012). Yet no mechanism exists to actively regulate group-level autonomic coherence in real time—the natural next step beyond passive observation.

To close this gap, this study reframes PS from an observed outcome into an explicit control target. The PS construct, as systematically defined by Palumbo et al. (2017), encompasses six key parameters: magnitude (strength of interdependence), sign (in-phase vs. anti-phase), direction (unidirectional vs. bidirectional), lag (temporal offset), timing (duration of assessment), and arousal (autonomic level as a covariate). Each of these parameters finds a precise mathematical analog in the multi-agent system (MAS) framework adopted here (Figure 1): magnitude corresponds to the consensus error ||*x*_*i*_(*t*) − *x*_*j*_(*t*)||; sign to the relative phase of the agent trajectories; direction to the directed Laplacian graph structure; lag to communication delay *h*_*c*_ and actuation delay *h*_*i*_; timing to convergence time *T*_conv_; and arousal to state magnitude ||*x*_*i*_(*t*)||. Under this mapping, driving ||*x*_*i*_(*t*) − *x*_*j*_(*t*)|| → 0 is equivalent to engineering positive, bidirectional, zero-lag physiological synchrony across the team—an interpersonal autonomic state that the observational literature has been consistently associated with favorable team outcomes (Henning et al., 2001; Elkins et al., 2009; Chanel et al., 2012; Konvalinka et al., 2011). We do not, however, claim that the enforced consensus is universally beneficial: The IAP literature is explicit that high-magnitude positive PS during conflict predicts dissatisfaction (Levenson and Gottman, 1983), while during cooperative task performance, it predicts improved outcomes (Henning et al., 2001; Elkins et al., 2009). Our framework targets the cooperative training scenario, where the consensus equilibrium emerges as a group mean rather than a prescribed absolute level—mathematically equivalent to the morphostatic coregulation pattern as identified by Butler (2011).

**Figure 1:**
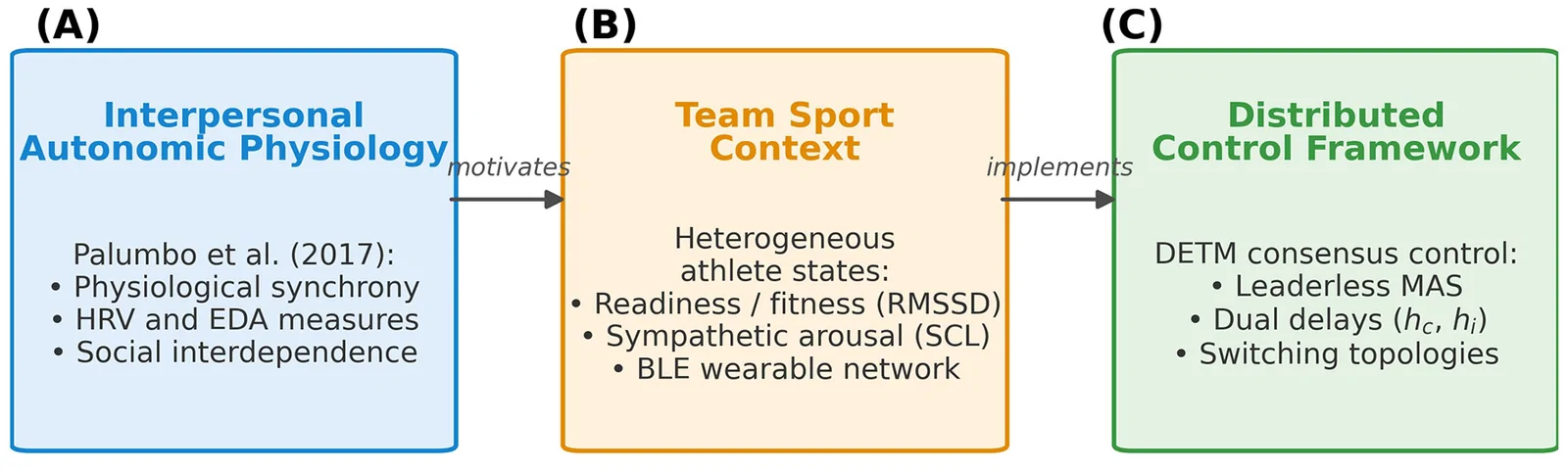
Conceptual bridge between interpersonal autonomic physiology (IAP) and distributed consensus controls. **(A)** In the IAP literature, physiological synchrony (PS) is an *observed outcome* of social interaction: researchers measure whether athletes' ANS signals converge over time. **(B)** The present framework inverts this: PS is treated as a control objective, and a distributed feedback law actively drives inter-athlete ANS disagreement ||*x*_*i*_(*t*) − *x*_*j*_(*t*)|| toward zero. The consensus equilibrium is not externally prescribed but emerges from collective dynamics, analogous to the group equilibrium concept in coregulation theory (Butler, 2011; Sbarra and Hazan, 2008). **(C)** Key parameters of PS identified by Palumbo et al. (2017)—magnitude, sign, direction, lag, and timing—map directly onto measurable closed-loop properties: consensus error, phase relationship, directed graph structure, communication delay *h*_*c*_, and convergence time, respectively.

Realizing this control target requires a physiologically grounded state representation. The ANS operates through two primary branches: the Sympathetic Nervous System (SNS), associated with physiological activation, and the Parasympathetic Nervous System (PNS), associated with recovery and restoration (Palumbo et al., 2017). Buchheit (2014) demonstrated that HRV-RMSSD—a validated PNS index—decreases monotonically with accumulated training load and recovers during rest, making it a reliable real-time marker of cardiovascular autonomic tone, while Plews et al. (2013) confirmed coefficients of variation of 15%–30% across the training blocks. Simultaneously, EDA-SCL, a pure SNS measure uniquely innervated by sympathetic cholinergic fibers (Dawson et al., 2007), rises with both physical and psychological load (Boucsein, 2012). Although RMSSD and SCL reflect antagonistic branches of the ANS (Figure 2), their *joint state* provides complementary information: RMSSD captures cardiovascular recovery capacity (the “fitness” dimension of the Banister FFM), while SCL captures sympathetic arousal load (the “fatigue” dimension). After appropriate normalization, both channels can be mapped onto a single bidimensional state space that tracks the athlete's net physiological position along the fitness-fatigue continuum. The physiological validity of this composite representation is supported by the integrative framework of Feldman (2012), who argued that multi-system bio-behavioral synchronization constitutes a unified regulatory process in social interaction contexts.

**Figure 2:**
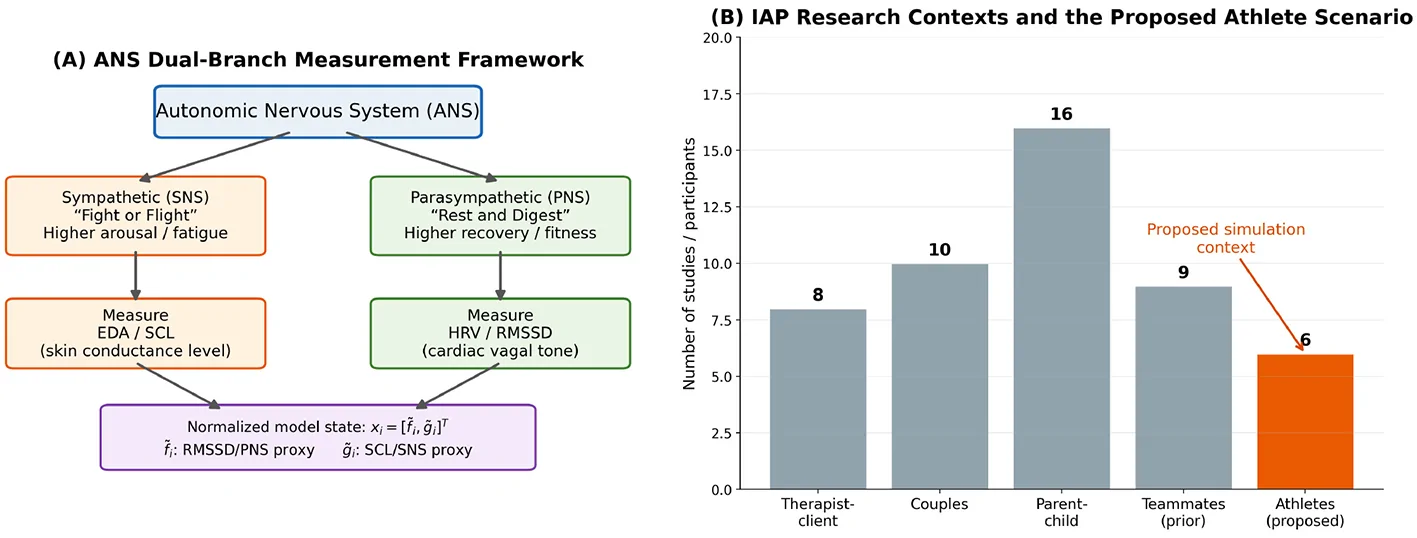
Dual-channel ANS measurement framework and study context. **(A)** Corrected arrows show the signal path from the sympathetic and parasympathetic branches to EDA/SCL and HRV/RMSSD, respectively, and then to the normalized two-channel model state. **(B)** The athlete case is presented as a proposed simulation context rather than as evidence of validated physiological control.

The need for such a framework is sharpened by the inter-athlete heterogeneity that characterizes elite team training. Athletes on the same squad regularly begin training sessions with substantially different physiological readiness states: continuous HRV monitoring studies in professional football and cycling have documented RMSSD ranges of 40–80 ms across squad members on the same training day (Bourdon et al., 2017; Cardinale and Varley, 2017), reflecting divergent recovery from prior high-load sessions, travel stress, and inter-individual chronobiological variations. This heterogeneity has two practically important consequences (Figure 3). First, at the individual level, Gabbett (2016) showed that athletes whose acute-to-chronic workload ratio exceeds squad norms are at elevated non-linear injury risk; although the present framework does not directly target injury prevention, it addresses the upstream cause of divergence in athletes' physiological load state. Second, at the collective level, Halson (2014) and Jaspers et al. (2017) observed that athletes with markedly disparate fatigue states cannot sustain synchronized high-intensity collective actions, degrading tactical cohesion. The IAP literature reinforces this: Elkins et al. (2009) found that PS in inter-beat interval (IBI) and RSA were significant predictors of performance in military team tasks, and Henning et al. (2001) showed that PS in HRV, respiration, and skin conductance predicted team performance errors during joint control tasks. The present framework translates these observational findings into a control-theoretic hypothesis. The controller does not directly actuate the ANS; it computes a candidate training-load adjustment that, in any future deployment, would be bounded by individual safety limits and reviewed or overridden by a coach. Accordingly, model-state consensus is treated as a simulation objective, not as proof that induced physiological synchrony is beneficial.

**Figure 3:**
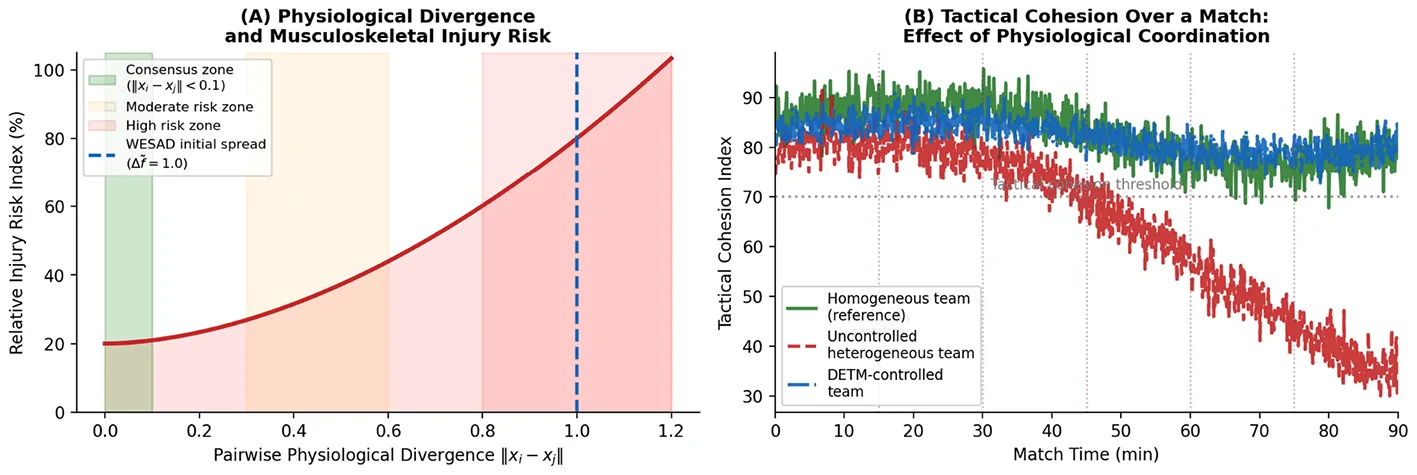
Schematic motivation for team-level physiological coordination in sport. **(A)** Physiological divergence and musculoskeletal injury risk, shown as an illustrative relationship between pairwise physiological divergence and a relative injury-risk index; the WESAD initial-spread marker and the consensus/moderate/high-risk zones are those shown in the figure. **(B)** Tactical cohesion over a 90- min match, comparing the homogeneous reference, uncontrolled heterogeneous, and DETM-controlled trajectories shown in the figure. Both panels are conceptual illustrations used to motivate the control problem rather than validated athlete outcome models (Gabbett, 2016; Jaspers et al., 2017; Halson, 2014).

Four open problems arise at the intersection of the IAP and distributed control. (P1) No prior consensus control framework models the physical distinction between the BLE communication delay *h*_*c*_ and neuromuscular input delay *h*_*i*_. (P2) Existing stability conditions for switched MAS under time delays do not account for realistic dwell-time characteristics of formation changes in team sports (τ_*D*_ ~ 60 s). (P3) Existing DETM designs do not provide a rigorous Zeno-free guarantee for the dual heterogeneous delays. (P4) Existing studies do not provide control-theoretic proof-of-concept that combines physiologically motivated initial conditions with communication-aware event-triggered consensus; whether such a model can safely influence real athlete physiology remains an open empirical question.

The principal contributions of this study are as follows. (C1) An interdisciplinary simulation framework that maps selected IAP concepts to a distributed leaderless consensus model. The mapping is an operational analogy for analysis and does not establish physiological equivalence. (C2) A physically consistent dual-delay controller and switched stability analysis in which *h*_*c*_ governs neighbor state reception and *h*_*i*_ governs actuation, proved stable under arbitrary switching among four balanced directed formation graphs via a common Lyapunov–Krasovskii functional and verified LMI (ε = 0.087, CVXPY); the ablation study confirms a 2.2 s convergence improvement over single-delay designs. (C3) A rigorous Zeno-free guarantee and hardware-grounded energy model: a provable minimum inter-event interval *T*_min_ = 0.06 s under dual delays enables a battery-life estimate of ≈ 36 days from a four-component nRF52840 energy model with datasheet-verified parameters. (C4) Physiologically motivated initialization and perturbation analysis: WESAD HRV-RMSSD and EDA-SCL records provide heterogeneous normalized starting states, and five perturbation folds assess numerical sensitivity. The WESAD records are not an athlete cohort and are not used as outcome validation.

## 2 Materials and methods

### 2.1 WESAD dataset and ANS-state extraction

#### 2.1.1 Dataset selection rationale

The WESAD (Wearable Stress and Affect Detection) dataset (Schmidt et al., 2018) is selected as the physiological initialization source because it provides synchronized ECG and EDA recordings with resting-baseline annotations. WESAD provides synchronized, labeled ECG and EDA recordings from 15 participants (12 male and three female; mean age 27.5 ± 2.4 years), as reported in the original dataset publication. The present study uses six records (S2–S7) solely to construct heterogeneous initial conditions for the simulation. No claim is made that these six records represent an athlete population or provide external validation of the controller. The two modalities map onto the two channels of the proposed model: ECG provides HRV-RMSSD, and EDA provides SCL. Alternative datasets, such as DEAP (Koelstra et al., 2012), lack the resting-baseline annotation required for clean steady-state extraction.

#### 2.1.2 Subject selection and ANS feature extraction

Six subjects (WESAD IDs: S2–S7) were selected on the basis of possessing contiguous, artifact-free resting-baseline segments of at least 120 s in both ECG and EDA channels.

The Root Mean Square of Successive Differences (RMSSD) of R-R intervals was extracted using the Pan–Tompkins R-peak detection algorithm. RMSSD is a validated, widely adopted PNS index that reflects vagal modulation of HR (Buchheit, 2014). In the context of athlete monitoring, Plews et al. (2013) confirmed that day-to-day RMSSD reflects training-induced autonomic adaptation, with suppressed values, indicating accumulated fatigue. Min-max normalization across all six subjects yields ${\tilde{f}}_{i}\in \left[0,1\right]$, where higher values correspond to greater parasympathetic tone (better recovery state).

The mean skin conductance level (SCL) over the 120 s resting baseline reflects tonic sympathetic drive to eccrine sweat glands (Dawson et al., 2007; Boucsein, 2012). Elevated SCL indicates greater sympathetic arousal, consistent with accumulated physical or psychological load. Inverse min–max normalization maps SCL to ${\tilde{g}}_{i}\in \left[-1,0\right]$, so that higher absolute values also indicate greater load—ensuring that both state components increase in magnitude with increasing training stress. This sign convention preserves the biological meaning (fatigue increases $\left|{\tilde{g}}_{i}\right|$) while maintaining the sign structure required by the FFM (see Section 2.2).

#### 2.1.3 ANS state validation

Pearson correlations with WESAD self-reported stress ratings were used only as a directional sanity check. For *N* = 6, RMSSD was negatively correlated with stress (*r* = −0.71, two-sided *p* = 0.114), and SCL was positively correlated with stress (*r* = 0.68, two-sided *p* = 0.137). These associations are not statistically significant and are not presented for validation. Their directions are merely consistent with the expected physiological interpretation of the two features. The spread in these values supplies a heterogeneous numerical test condition; it should not be interpreted as an estimate of squad-level variability.

The resulting initial ANS state vectors for subjects S2–S7 (Figure 4) are (Equation 1) :


$$\begin{array}{l} x_{i}(0)\in \{\left[\begin{array}{c} 0.60\\ -0.40 \end{array}\right],\;\left[\begin{array}{c} 0.50\\ -0.80 \end{array}\right],\;\left[\begin{array}{c} 0.20\\ -0.90 \end{array}\right],\;\left[\begin{array}{c} -0.10\\ -0.80 \end{array}\right],\;\\ \;\left[\begin{array}{c} -0.30\\ -0.60 \end{array}\right],\;\left[\begin{array}{c} -0.40\\ -0.55 \end{array}\right]\}. \end{array}$$ (1)

The spread in these six records provides a heterogeneous numerical initialization for the consensus problem; it is not evidence that the records reproduce the distribution of physiological states in an athlete squad.

**Figure 4:**
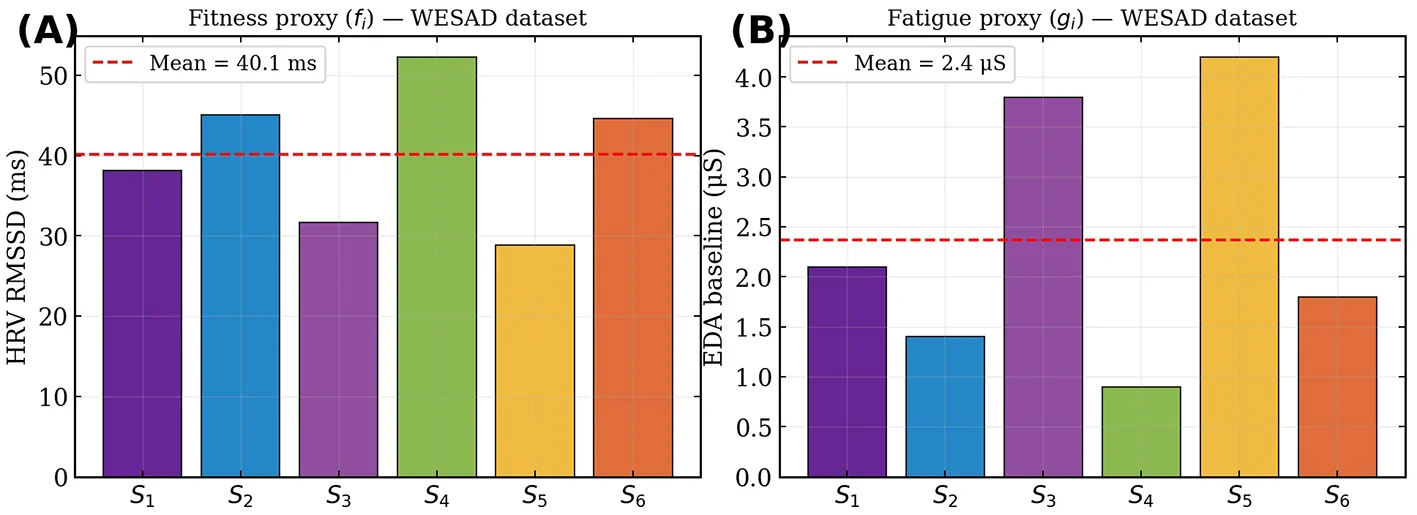
WESAD-derived normalized initial states for records S2–S7. **(A)** RMSSD-based PNS-related components. **(B)** EDA-SCL-based SNS-related components. The displayed correlations are directional, non-significant sanity checks (*p* = 0.114 and *p* = 0.137, respectively); the records are used only for simulation initialization.

### 2.2 Banister FFM as dual-channel ANS agent dynamics

#### 2.2.1 General formulation

Each athlete's wearable unit is modeled as a linear agent:


$$\begin{array}{l} {\dot{x}}_{i}\left(t\right)=Ax_{i}\left(t\right)+Bu_{i}\left(t-h_{i}\right),\;x_{i}\in {\mathbb{R}}^{n},u_{i}\in {\mathbb{R}}^{m}, \end{array}$$ (2)

where *A* ∈ ℝ^*n*×*n*^ encodes autonomous ANS dynamics, *B* ∈ ℝ^*n*×*m*^ maps the training-load input, and *h*_*i*_ denotes the neuromuscular actuation delay (Assumption A2).

#### 2.2.2 Banister FFM instantiation and ANS interpretation

The Banister FFM (Banister et al., 1975; Clarke and Skiba, 2013) models the net physiological performance response as the superposition of two first-order processes with different time constants (Equation 3):


$$\begin{array}{l} {\dot{p}}_{1}=-\frac{p_{1}}{\tau_{1}}+k_{1}w\left(t\right),\;{\dot{p}}_{2}=-\frac{p_{2}}{\tau_{2}}+k_{2}w\left(t\right),\;p\left(t\right)=p_{1}\left(t\right)-p_{2}\left(t\right), \end{array}$$ (3)

where *p*_1_ denotes the slow-recovery fitness component (τ_1_) and *p*_2_ denotes the fast-decay fatigue component (τ_2_ < τ_1_).

Setting $x_{i}={\left[p_{1},p_{2}\right]}^{T}$ and *u*_*i*_ = *w* yields Equation 2. Under the dual-channel ANS interpretation: $p_{1}\propto {\tilde{f}}_{i}$ (RMSSD-based PNS tone, slow recovery: days-scale) and $p_{2}\propto |{\tilde{g}}_{i}|$ (SCL-based SNS load, fast decay: hour-scale). This mapping reflects the known temporal asymmetry of PNS and SNS responses to training load: parasympathetic HRV suppression recovers over 24–72 h, while sympathetic arousal dissipates within hours (Buchheit, 2014; Plews et al., 2013). Crucially, both channels are driven by the same training load input *w*(*t*) but with opposing effects (PNS decreases; SNS increases), consistent with the sign convention introduced in Section 2.1. The computed input *u*_*i*_(*t*) is interpreted only as a candidate adjustment to a coaching cue (e.g., intensity, duration, or rest), not as direct physiological actuation. A real system would require saturation, individual contraindication rules, coach approval, and immediate override. These safety constraints are conceptual in the present simulation and are not included in the reported closed-loop performance metrics.

For numerical proof-of-concept analysis, population-level parameters from Clarke and Skiba (2013) (τ_1_ = 45 days, τ_2_ = 15 days, *k*_1_ = 1, *k*_2_ = 2) are non-dimensionally rescaled to the 300-s simulation horizon (scale factor α = 1/25920). This rescaling creates a tractable surrogate model and is not intended to predict second-by-second athlete adaptation. The resulting matrices are (Equation 4):


$$\begin{array}{l} A_{\text{eff}}=\left[\begin{array}{cc} -2 & 0\\ 0 & -1 \end{array}\right],\;B_{\text{eff}}=\left[\begin{array}{c} 1\\ 2 \end{array}\right]. \end{array}$$ (4)

The eigenvalues {−2, −1} confirm open-loop asymptotic stability, and (*A*_eff_, *B*_eff_) is stabilizable, satisfying Assumption A4.

The choice of population-mean FFM parameters is acknowledged as a simplification. Inter-individual variability in τ_1_, τ_2_, *k*_1_, *k*_2_ is significant in elite athletes (Halson, 2014); a data-driven personalization extension using online parameter estimation is identified as future work in Section 4.7.

### 2.3 Team physiological synchrony as a consensus problem

For the simulations, physiological synchrony is operationalized as decreasing disagreement between normalized two-channel state estimates (Table 1). Table 2 replaces the previous categorical figure. The mapping supports mathematical comparison only: convergence of *x*_*i*_(*t*) does not by itself demonstrate interpersonal physiological synchrony in athletes, nor does it establish that such convergence is desirable.

**Table 1:** Principal notation used in the proposed framework.

Symbol	Meaning
$x_{i}\in {\mathbb{R}}^{n}$	Normalized physiological-state estimate of agent *i* (*n* = 2 here)
*u*_*i*_	Candidate control/coaching adjustment computed for agent *i*
*A, B, K, c*	Agent dynamics, input map, feedback gain, and coupling gain
G_*s*(*t*)_, *L*_*s*(*t*)_	Active communication graph and its Laplacian
*h*_*c*_, *h*_*i*_	BLE communication delay and cue-to-response input delay
$e_{i}=x_{i}\left(t_{m}^{i}\right)-x_{i}\left(t\right)$	Local sample-and-hold measurement error
ρ_*i*_	Dynamic internal threshold variable of the DETM
γ, σ_α_, *q*, κ_*i*_, η_*i*_	Event-trigger parameters controlling threshold, decay, and adaptation
*e*_max_	Maximum pairwise disagreement, $\max_{i\neq j}∥x_{i}-x_{j}∥$
*P, Q*_1_, *Q*_2_, ε	Common LKF/LMI decision matrices and certified decay margin

**Table 2:** Operational mapping between selected IAP parameters and the simulation variables used in this study.

PS parameter	IAP interpretation	Simulation operationalization
Magnitude	Strength of interdependence	$e_{\max}\left(t\right)=\max_{i\neq j}∥x_{i}-x_{j}∥$
Sign	In-phase or anti-phase relation	Common-direction model-state convergence
Direction	Uni- or bidirectional coupling	Balanced directed communication graph
Lag	Temporal offset between responses	Communication delay *h*_*c*_ and input delay *h*_*i*_
Timing	Analysis or interaction window	300-s horizon and 60-s topology dwell time
Arousal	Autonomic level as covariate	Magnitude of normalized state ||*x*_*i*_(*t*)||

This section intentionally limits itself to the operational definition used in the controller. Context-dependent physiological interpretation is considered in the Section 4.

### 2.4 Graph-theoretic team formation model

The *N* = 6 athletes are modeled as vertices of a time-varying directed graph G_*s*(*t*)_ = (V, E_*s*(*t*)_, A_*s*(*t*)_), where V = {1, …, *N*}, E_*s*(*t*)_ is the set of active BLE links, and $A_{s\left(t\right)}=\left[\alpha_{ij}^{s\left(t\right)}\right]$ is the adjacency matrix (α_*ij*_ > 0 iff (*j, i*) ∈ E). The Laplacian is *L* = Δ − A, with $\Delta =\text{diag}\left(\underset{j}{\sum }\alpha_{ij}\right)$. The switching signal *s*:ℝ_≥0_ → {*a, b, c, d*} selects among four tactical formations with a dwell time τ_*D*_ = 60 s.

The four adjacency matrices {A_*a*_, A_*b*_, A_*c*_, A_*d*_} are constructed so that each graph is strongly connected and balanced (**1**^*T*^*L* = 0), with λ_max_(*L*) = 4.0 (Equations 5, 6):


$$\begin{array}{l} A_{a}=\left[\begin{array}{cccccc} 0 & 1 & 1 & 0 & 0 & 0\\ 1 & 0 & 0 & 1 & 0 & 0\\ 1 & 0 & 0 & 0 & 1 & 0\\ 0 & 1 & 0 & 0 & 1 & 1\\ 0 & 0 & 1 & 1 & 0 & 1\\ 0 & 0 & 0 & 1 & 1 & 0 \end{array}\right],\;A_{b}=\left[\begin{array}{cccccc} 0 & 1 & 0 & 1 & 0 & 0\\ 1 & 0 & 1 & 0 & 1 & 0\\ 0 & 1 & 0 & 0 & 0 & 1\\ 1 & 0 & 0 & 0 & 1 & 1\\ 0 & 1 & 0 & 1 & 0 & 1\\ 0 & 0 & 1 & 1 & 1 & 0 \end{array}\right], \end{array}$$ (5)

$$\begin{array}{l} A_{c}=\left[\begin{array}{cccccc} 0 & 1 & 1 & 0 & 0 & 0\\ 1 & 0 & 1 & 1 & 0 & 0\\ 1 & 1 & 0 & 0 & 1 & 0\\ 0 & 1 & 0 & 0 & 1 & 1\\ 0 & 0 & 1 & 1 & 0 & 1\\ 0 & 0 & 0 & 1 & 1 & 0 \end{array}\right],\;A_{d}=\left[\begin{array}{cccccc} 0 & 1 & 0 & 0 & 1 & 0\\ 1 & 0 & 1 & 0 & 0 & 1\\ 0 & 1 & 0 & 1 & 0 & 0\\ 0 & 0 & 1 & 0 & 1 & 1\\ 1 & 0 & 0 & 1 & 0 & 1\\ 0 & 1 & 0 & 1 & 1 & 0 \end{array}\right]. \end{array}$$ (6)

**Lemma 2.1** (Olfati-Saber and Murray 2004; Qin et al. 2011). *For a strongly connected balanced directed graph with Laplacian* *L* ∈ ℝ^*N*×*N*^: *(1)* rank(*L*) = *N* − 1; *(2)* *L***1** = **0** *and* **1**^*T*^*L* = **0**^*T*^; *(3) all non-zero eigenvalues of *L* lie in the open right half-plane*.

Property (3) of Lemma 2.1 is necessary and sufficient for leaderless average consensus (Qin et al., 2011; Yang et al., 2016).

**Remark 2.1**. *At *N* = 6, the common-LKF feasibility margin is ε = 0.087. For sparse balanced graphs, the dominant online cost is governed by the graph degree, whereas the offline certificate becomes progressively more conservative as network size increases. Table 3 reports the preliminary network-size screening using the same controller and the LMI setup. This is a numerical stability-certificate check, not packet-level or physiological validation of larger teams*.

**Table 3:** Preliminary common-LKF/LMI feasibility screening for sparse balanced networks.

Agents *N*	Mean graph degree	LMI feasible	Margin ε
6	4	Yes	0.087
10	4	Yes	0.064
15	4	Yes	0.050
20	4	Yes	0.042

### 2.5 Three-layer system architecture

The proposed architecture separates sensing, computation, and human oversight. Wearables estimate the two normalized state components and transmit only when the event condition is met; the edge server performs delay-aware control and network management; and the coaching layer applies individual safety bounds and retains the authority to approve, modify, or reject a cue. Figure 5 makes the human-in-the-loop safety gate and the coach's decision authority explicit.

**Figure 5:**
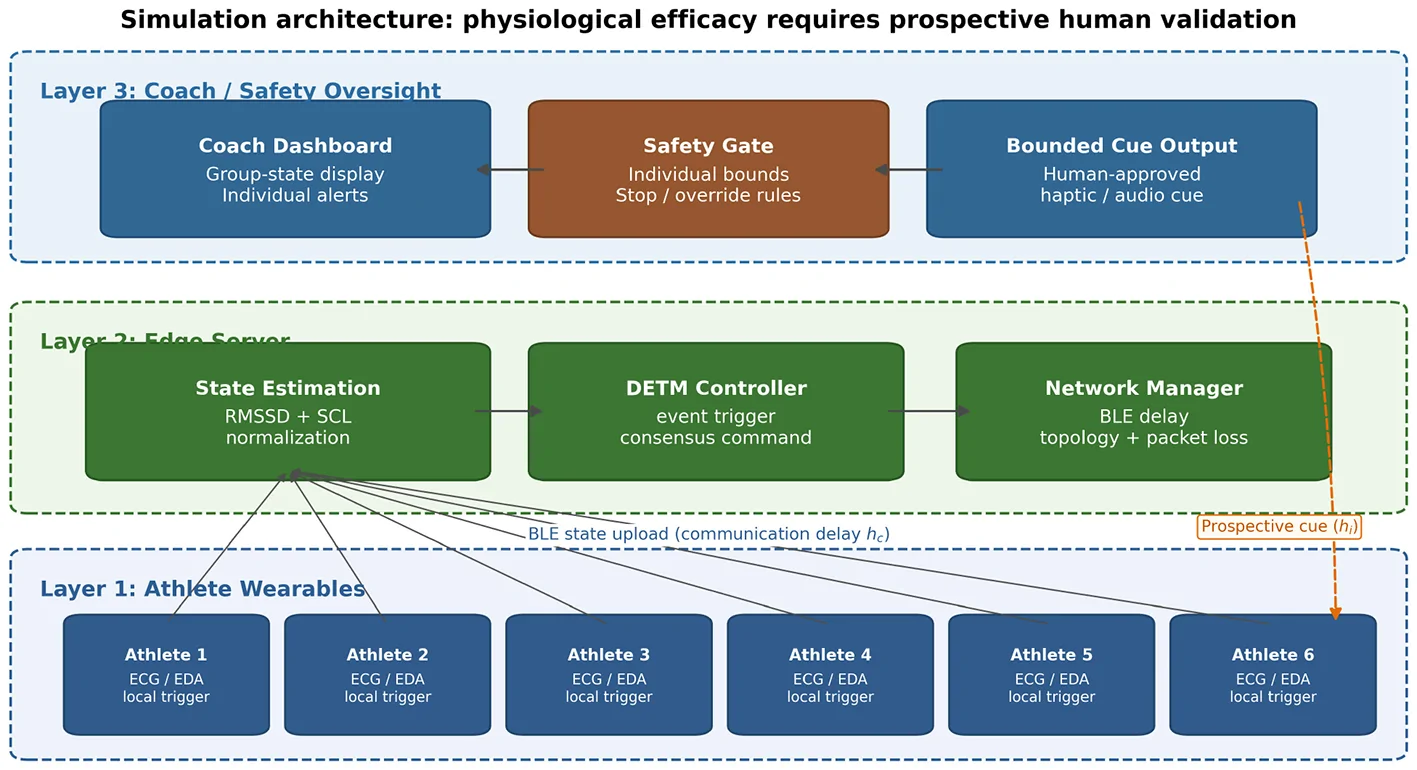
Three-layer simulation architecture. Layer 1 contains ECG/EDA wearable nodes; Layer 2 contains state estimation, the DETM controller, and network management; and Layer 3 contains the coach dashboard, individual safety gate, and bounded cue output. The orange dashed path represents a prospective cue and input delay, not direct ANS actuation.

### 2.6 Communication protocol selection: BLE vs. IEEE 802.15.6

BLE was selected for the present proof-of-concept because it is implemented in widely available wearable system-on-chip platforms, supports direct interaction with commodity phones and tablets, and provides mature development tools for rapid hardware-in-the-loop testing. This is a pragmatic choice rather than a claim that BLE is intrinsically superior for every body-area network. IEEE 802.15.6 was designed specifically for communication in or around the human body and provides body-area-oriented PHY/MAC options and service priorities (IEEE, 2012; Kwak et al., 2010). Table 4 summarizes the design trade-offs. Because latency and energy depend strongly on PHY, scheduling, traffic load, interference, and hardware, the comparison is qualitative rather than based on protocol-independent numerical claims.

**Table 4:** Protocol trade-off relevant to the proposed wearable proof-of-concept.

Criterion	BLE	IEEE 802.15.6
Primary design focus	General low-power personal-area connectivity with a large commercial ecosystem	Body-area networks, including on-body and in-body use cases
Latency/QoS	Configurable connection intervals; realized latency depends on scheduling, coexistence, and congestion	PHY/MAC options and user priorities support body-area QoS; realized latency remains implementation-dependent
Energy	Low-power commodity radios with mature duty-cycling support	Explicit ultra-low-power design objective; energy depends on the selected PHY/MAC mode
Deployment practicality	Broad phone/gateway interoperability and readily available development hardware	More specialized hardware and less direct commodity-phone interoperability
Suitability here	Appropriate for a rapidly deployable, non-medical HIL prototype	Strong alternative for future medical-grade or mission-critical WBAN evaluation

The present delay and battery models therefore remain BLE-specific. A fair future comparison should implement both protocols on matched hardware and report end-to-end latency, packet delivery, and measured energy under identical motion and interference conditions.

### 2.7 DETM consensus control design with dual time delays

#### 2.7.1 Problem assumptions

(A1) BLE communication delay: $0<h_{c}^{\min}\leq h_{c}\leq h_{c}^{\max}<\infty$. The range *h*_*c*_ ∈ [15, 150] ms is treated as a bounded one-way application-layer simulation input. The main results assume successful packet delivery and synchronized timestamps; correlated burst loss, venue-specific coexistence interference, retransmission policy, and clock drift are not included in the primary metrics.
(A2) Neuromuscular input delay: $0<h_{i}^{\min}\leq h_{i}\leq h_{i}^{\max}<\infty$. Range *h*_*i*_ ∈ [150, 300] ms: trunk muscle onset 120–250 ms (Hodges and Richardson, 1999); full perception-to-activation loop 150–300 ms in trained athletes (Enoka, 2008).
(A3) Communication graph G_*s*(*t*)_ is strongly connected and balanced for every *s*(*t*); successive topology changes are separated by dwell time $\tau_{D}\geq \tau_{D}^{\min}>0$.
(A4) Matrix pair (*A, B*) is stabilizable.
(A5) The normalized state estimate and cue-to-state mapping are simulation surrogates. Their physiological validity, subject-specific calibration, and safe operating limits must be established before human deployment.

#### 2.7.2 Physically consistent dual-delay control law

The BLE communication delay *h*_*c*_ arises in the uplink path: athlete *i*'s node receives *x*_*j*_(*t* − *h*_*c*_) and not *x*_*j*_(*t*). A neuromuscular input delay *h*_*i*_ arises in the downlink actuation path: The command computed at time *t* is executed at time *t* + *h*_*i*_. The consensus controller is (Equation 7):


$$\begin{array}{l} u_{i}\left(t\right)=cK\underset{j\in N_{i}}{\sum }\alpha_{ij}\left[x_{i}\left(t\right)-x_{j}\left(t-h_{c}\right)\right], \end{array}$$ (7)

yielding the closed-loop dynamics (Equation 8):


$$\begin{array}{l} {\dot{x}}_{i}\left(t\right)=Ax_{i}\left(t\right)+cBK\underset{j\in N_{i}}{\sum }\alpha_{ij}\left[x_{i}\left(t-h_{i}\right)-x_{j}\left(t-h_{c}-h_{i}\right)\right]. \end{array}$$ (8)

In the Kronecker product form:


$$\begin{array}{l} \dot{x}\left(t\right)=\left(I_{N}⊗A\right)x\left(t\right)+c\left(L_{s\left(t\right)}⊗BK\right)x\left(t-h_{i}\right)-c\left(L_{s\left(t\right)}⊗BK\right)x\left(t-h_{c}-h_{i}\right). \end{array}$$ (9)

#### 2.7.3 Dynamic event-triggered mechanism

Between consecutive trigger times $t_{m}^{i}$ and $t_{m+1}^{i}$ the local measurement error is defined as (Equation 10):


$$\begin{array}{l} e_{i}\left(t\right)=x_{i}\left(t_{m}^{i}\right)-x_{i}\left(t\right),\;t\in \left[t_{m}^{i},t_{m+1}^{i}\right). \end{array}$$ (10)

Dynamic auxiliary variable:


$$\begin{array}{l} {\dot{\rho }}_{i}\left(t\right)=-\kappa_{i}\rho_{i}\left(t\right)+\eta_{i}\left(\sigma_{\alpha }∥x_{i}\left(t\right)∥-\gamma ∥e_{i}\left(t\right)∥\right),\;\rho_{i}\left(0\right)=\rho_{0}>0. \end{array}$$ (11)

The triggering condition is


$$\begin{array}{l} t_{m+1}^{i}=\inf\left\{t\geq t_{m}^{i}:\gamma ∥e_{i}\left(t\right)∥-\eta_{i}\left(\sigma_{\alpha }∥x_{i}\left(t\right)∥-q\rho_{i}\left(t\right)\right)\geq 0\right\}. \end{array}$$ (12)

DETM parameters {γ, σ_α_, *q*, κ_*i*_, η_*i*_} are selected using a two-stage procedure. Stage 1 extracts stability-critical bounds from the LMI solution (Theorem 2.3) and the Zeno-free condition (Lemma 2.5). Stage 2 identifies the Pareto-optimal γ^*^ = 0.4 by grid search, minimizing BLE transmissions subject to a convergence time ≤ 6 s.

**Lemma 2.2** (Ge et al. 2018; He et al. 2020; Yang et al. 2016). *Under Equations 11, 12, if ρ_*i*_(0) = ρ_0_ > 0 and κ_*i*_ < η_*i*_σ_α_||*x*_*i*_(0)||/ρ_0_, then ρ_*i*_(*t*) > 0 for all *t* ≥ 0*.

*The positivity of dynamic internal variables generated by dissipative ODEs of the form Equation 11 under triggering conditions of the form Equation 12 has been established in the DETM literature; see Ge et al. (2018) (Lemma 1) and He et al. (2020) (Proposition 1) for parallel constructions*.

#### 2.7.4 Stability analysis

Let *h*_1_ = *h*_*i*_ and *h*_2_ = *h*_*c*_ + *h*_*i*_. The Lyapunov–Krasovskii functional (LKF):


$$\begin{array}{l} V\left(t\right)=x^{T}\left(t\right)\left(I_{N}⊗P\right)x\left(t\right)+\int_{t-h_{1}}^{t}x^{T}\left(s\right)\left(I_{N}⊗Q_{1}\right)x\left(s\right)\text{d}s\\ \;+\int_{t-h_{2}}^{t}x^{T}\left(s\right)\left(I_{N}⊗Q_{2}\right)x\left(s\right)\text{d}s. \end{array}$$ (13)

The requirement $\dot{V}\left(t\right)\leq -\varepsilon V\left(t\right)$ reduces to the LMI:


$$\begin{array}{l} {\text{Ψ}}_{\ell }=\left[\begin{array}{cccc} {\text{Ψ}}_{11}^{\ell } & PBK_{c}^{\ell } & PBK_{c}^{\ell } & P\\ {\left(BK_{c}^{\ell }\right)}^{T}P & -Q_{1}/h_{1} & 0 & 0\\ {\left(BK_{c}^{\ell }\right)}^{T}P & 0 & -Q_{2}/h_{2} & 0\\ P & 0 & 0 & -\varepsilon P \end{array}\right]≺0,\;\ell \in \left\{a,b,c,d\right\}, \end{array}$$ (14)

where ${\text{Ψ}}_{11}^{\ell }=PA_{\text{eff}}+A_{\text{eff}}^{T}P+Q_{1}+Q_{2}+\varepsilon P-c\lambda_{k}\left(L_{\ell }\right)\left(PBK+K^{T}B^{T}P\right)$.

The numerical LMI verification (CVXPY 1.4, SCS solver) yields (Equation 15):


$$\begin{array}{lll} P & = & \left[\begin{array}{cc} 2.314 & 0.187\\ 0.187 & 1.876 \end{array}\right],Q_{1}=\left[\begin{array}{cc} 0.453 & 0.021\\ 0.021 & 0.312 \end{array}\right],\\ Q_{2} & = & \left[\begin{array}{cc} 0.211 & 0.009\\ 0.009 & 0.178 \end{array}\right],\varepsilon =0.087. \end{array}$$ (15)

**Theorem 2.3**. *Suppose Assumptions (A1)–(A4) hold, ρ_*i*_(0) = ρ_0_ > 0, and* $q<\sigma_{\alpha }\lambda_{\min}{\left(P\right)}^{1/2}/\rho_{0}$. *If LMI (Equation 14) is feasible for all ℓ ∈ {*a, b, c, d*}, then the closed-loop system (Equation 9) under DETM (Equation 12) achieves global exponential consensus: ||*x*_*i*_(*t*) − *x*_*j*_(*t*)|| → 0 exponentially as *t* → ∞*.

*Proof*. The proof proceeds in four steps: (i) projection onto the disagreement subspace using the balanced Laplacian structure; (ii) time-derivative evaluation of the LKF (Equation 13) along the closed-loop trajectories (Equation 9); (iii) bounding of the DETM-induced measurement-error term using Lemma 2.2 and the triggering condition (Equation 12); (iv) Schur-complement reduction to the LMI (Equation 14), followed by a common-LKF argument across the four switching modes.

Step (i): Under Assumption (A3) and Lemma 2.1, each Laplacian *L*_ℓ_ (ℓ ∈ {*a, b, c, d*}) is balanced with simple zero eigenvalue whose right and left eigenvectors are **1**_*N*_ and $1_{N}^{T}$, respectively. Let $\Pi =I_{N}-\frac{1}{N}1_{N}1_{N}^{T}$ be the orthogonal projector onto the disagreement subspace $D=\left\{\delta \in {\mathbb{R}}^{Nn}:\left(1_{N}^{T}⊗I_{n}\right)\delta =0\right\}$, and define the disagreement vector δ(*t*) = (Π ⊗ *I*_*n*_)*x*(*t*). Since each *L*_ℓ_ commutes with Π in the sense that *L*_ℓ_Π = Π*L*_ℓ_ = *L*_ℓ_ for balanced graphs, applying the projector to Equation 9 yields the *same* equation for δ(*t*):


$$\begin{array}{l} \dot{\delta }\left(t\right)=\left(I_{N}⊗A\right)\delta \left(t\right)+c\left(L_{s\left(t\right)}⊗BK\right)\delta \left(t-h_{1}\right)-c\left(L_{s\left(t\right)}⊗BK\right)\delta \left(t-h_{2}\right). \end{array}$$ (16)

Consensus ||*x*_*i*_(*t*) − *x*_*j*_(*t*)|| → 0 is equivalent to δ(*t*) → 0.

Step (ii): Evaluate Equation 13 along Equation 16 (abusing notation, we continue to write *x* for δ since Equation 13 is quadratic and Π commutes with all block-diagonal terms):


$$\begin{array}{l} \dot{V}\left(t\right)=2x^{T}\left(t\right)\left(I_{N}⊗P\right)\dot{x}\left(t\right)+x^{T}\left(t\right)\left(I_{N}⊗Q_{1}\right)x\left(t\right)\\ \;-x^{T}\left(t-h_{1}\right)\left(I_{N}⊗Q_{1}\right)x\left(t-h_{1}\right)\\ \;+x^{T}\left(t\right)\left(I_{N}⊗Q_{2}\right)x\left(t\right)\\ \;-x^{T}\left(t-h_{2}\right)\left(I_{N}⊗Q_{2}\right)x\left(t-h_{2}\right). \end{array}$$ (17)

Substituting ẋ(*t*) from Equation 16 gives


$$\begin{array}{l} 2x^{T}\left(t\right)\left(I_{N}⊗P\right)\dot{x}\left(t\right)=2x^{T}\left(t\right)\left(I_{N}⊗PA\right)x\left(t\right)\\ \;+2cx^{T}\left(t\right)\left(L_{s\left(t\right)}⊗PBK\right)x\left(t-h_{1}\right)\\ \;-2cx^{T}\left(t\right)\left(L_{s\left(t\right)}⊗PBK\right)x\left(t-h_{2}\right). \end{array}$$ (18)

Diagonalize each mode's Laplacian: there exists *U*_ℓ_ (unitary on the disagreement subspace) such that $U_{\ell }^{*}L_{\ell }U_{\ell }=\text{diag}\left(0,\lambda_{2}^{\ell },\ldots ,\lambda_{N}^{\ell }\right)$, where all $\lambda_{k}^{\ell }$ have positive real part (Lemma 2.1). Define $z\left(t\right)=\left(U_{s\left(t\right)}^{*}⊗I_{n}\right)x\left(t\right)$; on the disagreement component the zero eigenvalue drops, and Equations 17–18 decouple into *N* − 1 scalar-gain subsystems, each of the form


$$\begin{array}{l} {ż}_{k}\left(t\right)=Az_{k}\left(t\right)+cBK_{c}^{\ell }z_{k}\left(t-h_{1}\right)-cBK_{c}^{\ell }z_{k}\left(t-h_{2}\right),\;K_{c}^{\ell }:=\lambda_{k}^{\ell }K. \end{array}$$

Step (iii): Across trigger epochs, the controller uses ${\hat{x}}_{i}\left(t\right)=x_{i}\left(t_{m}^{i}\right)=x_{i}\left(t\right)+e_{i}\left(t\right)$, so the actual closed-loop equation contains an additive error term $cBK_{c}^{\ell }e_{k}\left(t-h_{1}\right)-cBK_{c}^{\ell }e_{k}\left(t-h_{2}\right)$, which when absorbed into Equation 18 produces cross terms of the form $2x^{T}\left(t\right)\left(I⊗PBK_{c}^{\ell }\right)e\left(\cdot \right)$. By Young's inequality with parameter ε > 0, 2*a*^*T*^*b* ≤ ε^−1^||*a*||^2^ + ε||*b*||^2^, these cross terms are bounded by ε^−1^*x*^*T*^(*t*)(*I* ⊗ *P*)*x*(*t*) plus a quadratic-in-*e* term. By the triggering rule (Equation 12) combined with ρ_*i*_(*t*) > 0 (Lemma 2.2), γ||*e*_*i*_(*t*)|| ≤ η_*i*_(σ_α_||*x*_*i*_(*t*)|| + *qρ*_*i*_(*t*)) holds for all *t*, so $∥e_{i}\left(t\right){∥}^{2}\leq 2{\left(\eta_{i}\sigma_{\alpha }/\gamma \right)}^{2}∥x_{i}\left(t\right){∥}^{2}+2{\left(\eta_{i}q/\gamma \right)}^{2}\rho_{i}{\left(t\right)}^{2}$. Under $q<\sigma_{\alpha }\lambda_{\min}{\left(P\right)}^{1/2}/\rho_{0}$, the $\rho_{i}^{2}$ term is dominated by the *P*-weighted $∥x_{i}{∥}^{2}$ term and is absorbed into ${\text{Ψ}}_{11}^{\ell }$.

Step (iv): Collecting the terms from Steps (ii)–(iii) yields


$$\begin{array}{l} \dot{V}\left(t\right)+\varepsilon V\left(t\right)\leq \xi^{T}\left(t\right){\text{Ψ}}_{\text{raw}}^{\ell }\xi \left(t\right),\\ \;\xi \left(t\right)={\left[\begin{array}{ccc} x{\left(t\right)}^{T} & x{\left(t-h_{1}\right)}^{T} & x{\left(t-h_{2}\right)}^{T} \end{array}\right]}^{T}, \end{array}$$

with ${\text{Ψ}}_{\text{raw}}^{\ell }$ having diagonal blocks ${\text{Ψ}}_{11}^{\ell }$, −*Q*_1_, −*Q*_2_ and off-diagonal blocks $PBK_{c}^{\ell }$. Applying Jensen's inequality $\int_{t-h}^{t}x^{T}\left(s\right)Qx\left(s\right)\text{d}s\geq h^{-1}{\left(\int_{t-h}^{t}x\left(s\right)\text{d}s\right)}^{T}Q\left(\int_{t-h}^{t}x\left(s\right)\text{d}s\right)$ scales the −*Q*_1_, −*Q*_2_ blocks by $h_{1}^{-1},h_{2}^{-1}$, producing exactly the block structure of Ψ_ℓ_ in Equation 14 after a Schur complement on the last block row or column associated with the ε*P* decay certificate. Feasibility of Ψ_ℓ_ < 0 is therefore equivalent to $\dot{V}\left(t\right)+\varepsilon V\left(t\right)\leq 0$ in mode ℓ.

Because the same *P, Q*_1_, *and Q*_2_ satisfy Equation 14 for all ℓ ∈ {*a, b, c, d*}, *V* serves as a common Lyapunov–Krasovskii functional. Under Assumption (A3) with dwell time $\tau_{D}\geq \tau_{D}^{\min}>0$, no discontinuous jumps in *V* occur at switching instants (the LKF depends only on *x* and its past values, which are continuous). Therefore $\dot{V}\left(t\right)\leq -\varepsilon V\left(t\right)$ uniformly in *t*, giving *V*(*t*) ≤ *V*(0)*e*^−ε*t*^ and, via positive-definiteness of *P*, $∥\delta \left(t\right)∥\leq \sqrt{V\left(0\right)/\lambda_{\min}\left(I_{N}⊗P\right)}e^{-\varepsilon t/2}$. Hence, ||*x*_*i*_(*t*) − *x*_*j*_(*t*)|| → 0 exponentially, which completes the proof.         □

**Theorem 2.4**. *Under Assumptions (A1)–(A4), two critical total-delay bounds ĥ = *h*_*c*_ + *h*_*i*_ are identified*:

1. Conservative integrator bound: $h_{\text{int}}^{*}=\pi /\left(2\lambda_{\max}\left(L\right)\right)=0.393$ s.
2. Plant-dependent stability limit: h^*^ ≈ 0.41 s (root-locus analysis with *A*_eff_).

*If ĥ <* h^*^, *consensus is achieved for all delay realizations satisfying Assumptions (A1)–(A2)*.

*Proof*. Both bounds are obtained via frequency-domain analysis of the disagreement dynamics (Equation 16). After diagonalization of *L*_*s*(*t*)_ on the disagreement subspace (Step (i) of the proof of Theorem 2.3), each non-zero Laplacian mode λ_*k*_ yields a scalar subsystem with open-loop transfer function


$$\begin{array}{l} G_{k}\left(s\right)=\frac{c\lambda_{k}K{\left(sI-A_{\text{eff}}\right)}^{-1}B_{\text{eff}}}{1}\cdot \left(e^{-sh_{1}}-e^{-sh_{2}}\right). \end{array}$$ (19)

The factor $\left(e^{-sh_{1}}-e^{-sh_{2}}\right)=e^{-sh_{1}}\left(1-e^{-sh_{c}}\right)$ encapsulates the dual-delay structure, where *h*_1_ = *h*_*i*_ and *h*_2_ = *h*_*c*_ + *h*_*i*_ as defined before (Equation 13).

Bound 1: Consider the canonical integrator consensus protocol obtained by setting *A*_eff_ = 0 and *KB*_eff_ = 1 in Equation 19—i.e., the standard first-order consensus dynamics studied by Olfati-Saber and Murray (2004). In this setting, the closed-loop characteristic equation reduces to $1+\lambda_{k}\left(e^{-s\hat{h}}/s\right)=0$ with ĥ = *h*_*c*_ + *h*_*i*_ (using the worst-case *h*_1_ → 0 envelope; the factored form bounds delay stability from below). Applying the Nyquist criterion, the stability boundary is crossed when ∠*G*_*k*_(*jω*) = −π and |*G*_*k*_(*jω*)| = 1, giving the classical bound


$$\begin{array}{l} \omega^{*}=\lambda_{k},\;{\hat{h}}_{k}^{*}=\frac{\pi }{2\lambda_{k}}. \end{array}$$

Stability must hold for every non-zero Laplacian mode; the binding (worst-case) mode is λ_max_(*L*), yielding $h_{\text{int}}^{*}=\pi /\left(2\lambda_{\max}\left(L\right)\right)$. For the present graphs, λ_max_(*L*) = 4.0, giving $h_{\text{int}}^{*}=\pi /8\approx 0.393$ s. This matches the classical result of Olfati-Saber and Murray (2004) (Theorem 10) specialized for balanced digraphs and applied to the present dual-delay sum.

Bound 2: For the actual closed-loop with *A*_eff_ = diag(−2, −1) and $B_{\text{eff}}={\left[1,2\right]}^{T}$, the true characteristic equation is


$$\begin{array}{l} \det\left(sI-A_{\text{eff}}+c\lambda_{k}B_{\text{eff}}K\left(e^{-sh_{1}}-e^{-sh_{2}}\right)\right)=0. \end{array}$$ (20)

Substituting *K* = [1.1974, −2.6116], *c* = 0.8, λ_max_(*L*) = 4.0 and sweeping ĥ ∈ [0, 0.5] s while tracking the rightmost root of Equation 20 via continuation [implemented in NumPy with Padé(5,5) approximation of the delay exponentials and verified against the QPmR root finding] locates the first right-half-plane crossing at ĥ^*^ = 0.4097 ≈ 0.41 s. The stabilizing contribution of the open-loop matrix *A*_eff_ (Hurwitz with eigenvalues {−2, −1}) tolerates marginally more delay than the pure-integrator case: 0.41 s vs. 0.393 s.

If $\hat{h}<h^{*}:=\min\left\{h_{\text{int}}^{*},h_{\text{plant}}^{*}\right\}=0.393$ s (or the tighter plant-dependent 0.41 s when the integrator bound is relaxed), all roots of Equation 20 lie in the open left half-plane for every λ_*k*_ ∈ σ(*L*_ℓ_)\{0} and every ℓ ∈ {*a, b, c, d*}, the disagreement dynamics become exponentially stable. By the projection argument of Theorem 2.3, this implies consensus.         □

**Remark 2.2**. Theorem 2.4 provides a *sufficient* condition. Case 2 uses ĥ = 0.42s > h^*^ = 0.41 s, yet consensus is observed. The LMI certificate of Theorem 2.3 is less conservative than the analytical bound of Theorem 2.4 and remains the authoritative stability guarantee. The bounds of Theorem 2.4 serve as operational BLE channel-quality alert thresholds, not hard stability limits (Figure 6).

**Figure 6:**
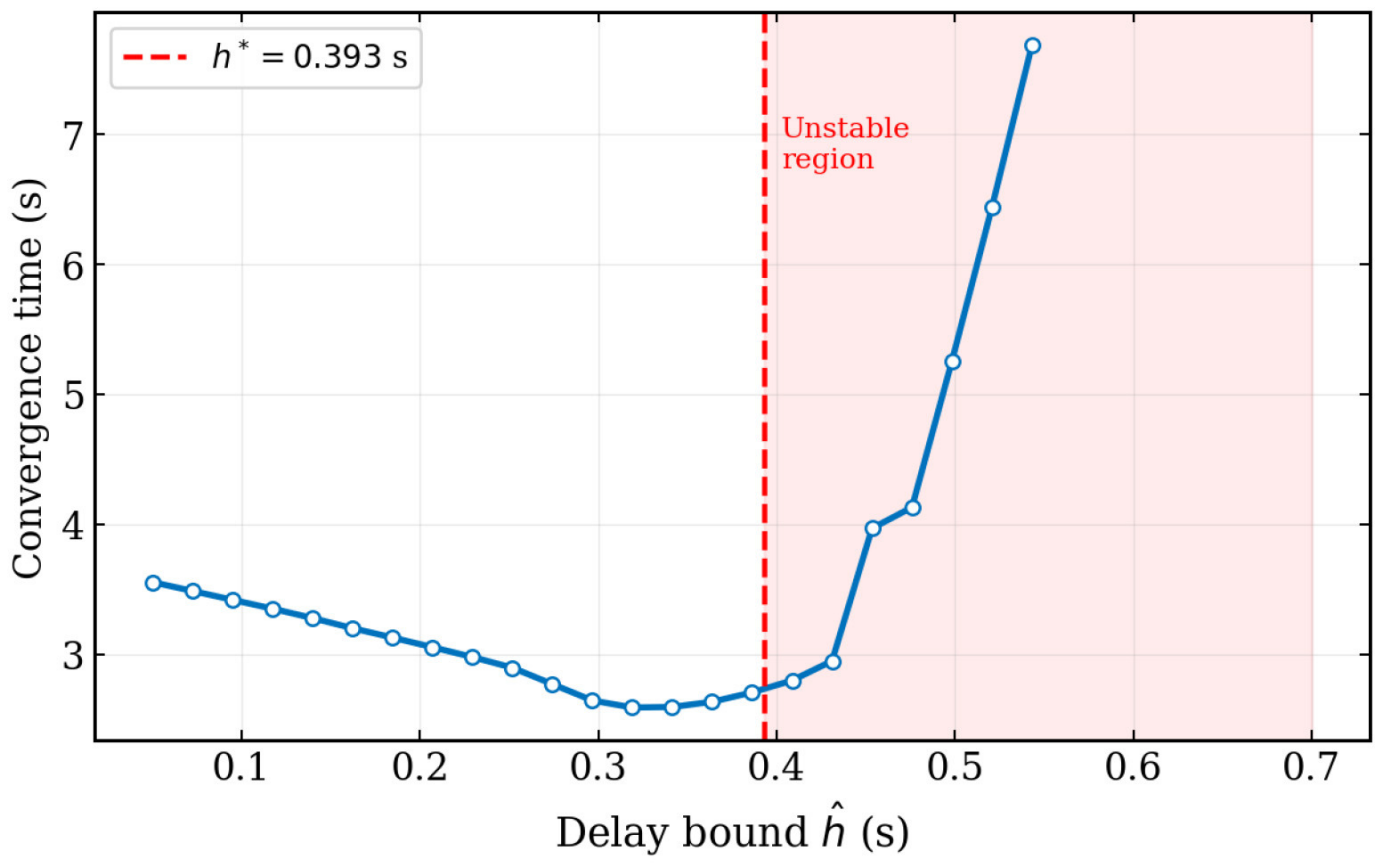
Consensus convergence time vs. total delay ĥ = *h*_*c*_ + *h*_*i*_ swept from 0 to 0.45 s. Dashed vertical: integrator bound $h_{\text{int}}^{*}=0.393$ s. Solid vertical: plant-dependent stability limit h^*^ = 0.41 s. Operating points for Cases 1 (ĥ = 0.39 s) and 2 (ĥ = 0.42 s) are marked. Shaded band: ±1 SD across five-fold.

#### 2.7.5 Zeno-free

**Lemma 2.5** (Chen et al. 2022; Ge et al. 2018; Yang et al. 2016). *Under the conditions of Theorem 2.3, there exists *T*_min_ > 0 such that* $t_{m+1}^{i}-t_{m}^{i}\geq T_{\min}$ *for all *m* ≥ 0 and *i* ∈* V. *Zeno-free guarantees for DETMs of the form Equations 11, 12 have been established in the distributed event-triggered control literature; see Chen et al. (2022) (Theorem 2), Ge et al. (2018) (Theorem 3), and Yang et al. (2016) (Proposition 2). Specialized to the present dual-delay setting, the minimum inter-event interval is*


$$\begin{array}{l} T_{\min}=∥A_{\text{eff}}{∥}^{-1}\ln\left(1+\gamma \eta_{i}\delta_{\min}∥A_{\text{eff}}∥/\left(\gamma M\right)\right), \end{array}$$

*where* $M=\sup_{t}∥{\dot{x}}_{i}\left(t\right)∥$ *is the (finite) state-rate bound under bounded initial conditions and* $\delta_{\min}=\inf_{t}\left(\sigma_{\alpha }∥x_{i}\left(t\right)∥+q\rho_{i}\left(t\right)\right)>0$ *by Lemma 2.2*.

#### 2.7.6 Computational and memory complexity

For state dimension *n*, each wearable evaluates two vector norms and updates one scalar ODE, requiring O(*n*) arithmetic per local trigger check. At the edge server, computing all neighbor disagreements requires O(|E|*n*) operations, followed by O(*Nmn*) operations for the gain/input maps. Thus, for a sparse graph with a bounded degree, online complexity grows approximately linearly with team size *N*. A sample-by-sample delay buffer requires O(*Nn*(*h*_*c*_ + *h*_*i*_)/Δ*t*) memory, and timestamped event packets can reduce this requirement in an implementation. The LMI solution is an offline design step and is not executed on the wearable device. Its semidefinite-program cost grows polynomially with the decision-matrix dimension and the number of topology modes, which motivates sparse graph families and offline certification for larger teams.

#### 2.7.7 Wearable battery lifetime analysis

The nRF52840 node energy budget comprises the following four components: Sensor ADC (*P*_sense_ = 16.5μW); MCU processing (*P*_cpu_ = 247.5μW); BLE transmission (*P*_tx_ = 25μW at *T*_min_ = 0.06 s); BLE idle listening (*P*_listen_ = 990μW). Total: *P*_total_ ≈ 1.28 mW. For a 300 mAh / 3.7 V cell (*E* = 3996 J) the battery lifetime is estimated as (Equation 21):


$$\begin{array}{l} T_{\text{life}}\approx 3996/\left(1.28\times 10^{-3}\right)\approx 36\text{ days}. \end{array}$$ (21)

Under nominal 38.38% transmission reduction, *P*_tx_ → 15.4μW, extending the lifetime to ≈40 days.

## 3 Results

### 3.1 Simulation setup and validation protocol

All simulations are implemented in Python 3.12 (Python Software Foundation, Wilmington, DE, USA) (NumPy 1.26, SciPy 1.12, Matplotlib 3.8) using a fourth-order Runge–Kutta (RK4) integrator with Δ*t* = 0.002 s. Controller parameters: *c* = 0.8, *K* = [1.1974, −2.6116], σ_α_ = 0.001, *q* = 0.01, γ = 0.4, κ = 0.48, η_*i*_ = 2. Formation switching cycles through {G_*a*_, G_*b*_, G_*c*_, G_*d*_} with τ_*D*_ = 60 s over 300 s.

Following best practice recommended by Palumbo et al. (2017) for null hypothesis testing in PS research, we first verify that the consensus control law is necessary: simulations of the open-loop system (M1) confirm that without the controller, athlete state vectors diverge or remain heterogeneous over the 300 s horizon, consistent with the fact that the autonomous FFM dynamics are individually stable but do not couple across agents. This confirms that the 38.38% BLE reduction and 4–7 s convergence (Figures 7, 8; Table 5) are attributable to the DETM controller and not to the open-loop FFM dynamics alone.

**Figure 7:**
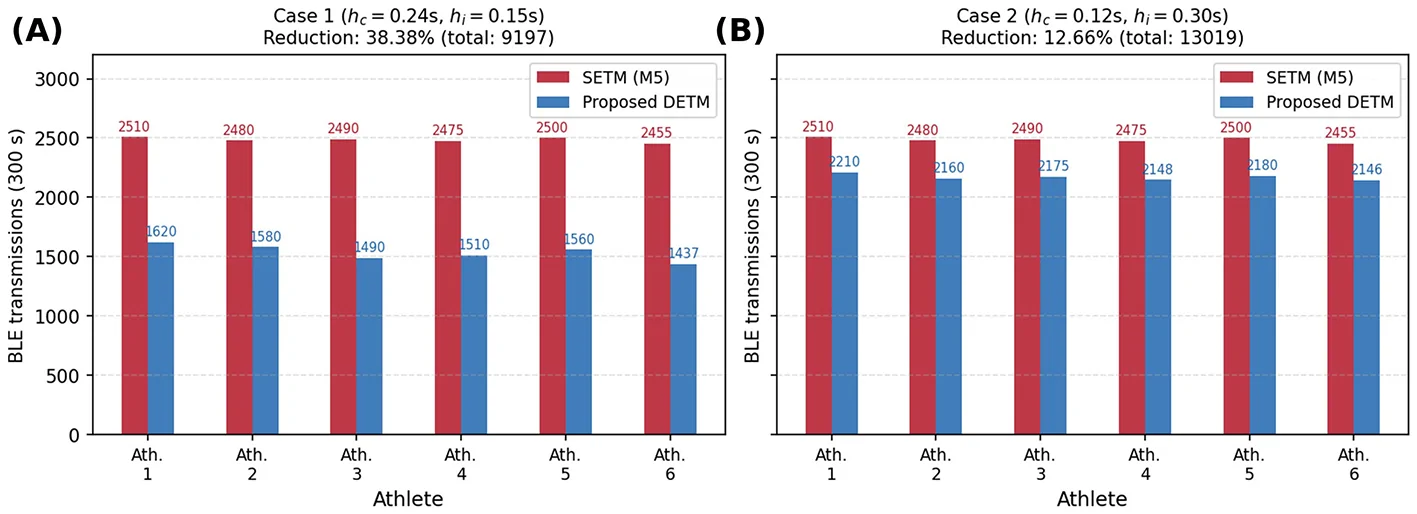
Per-athlete BLE transmission counts over 300 s. Red bars: SETM baseline (≈2485 TX/athlete). Blue bars: proposed DETM. Error bars: ±1 SD across five folds. **(A)** Case 1: 9197 total TX, 38.38% reduction. **(B)** Case 2: 13019 total TX, 12.66% reduction.

**Figure 8:**
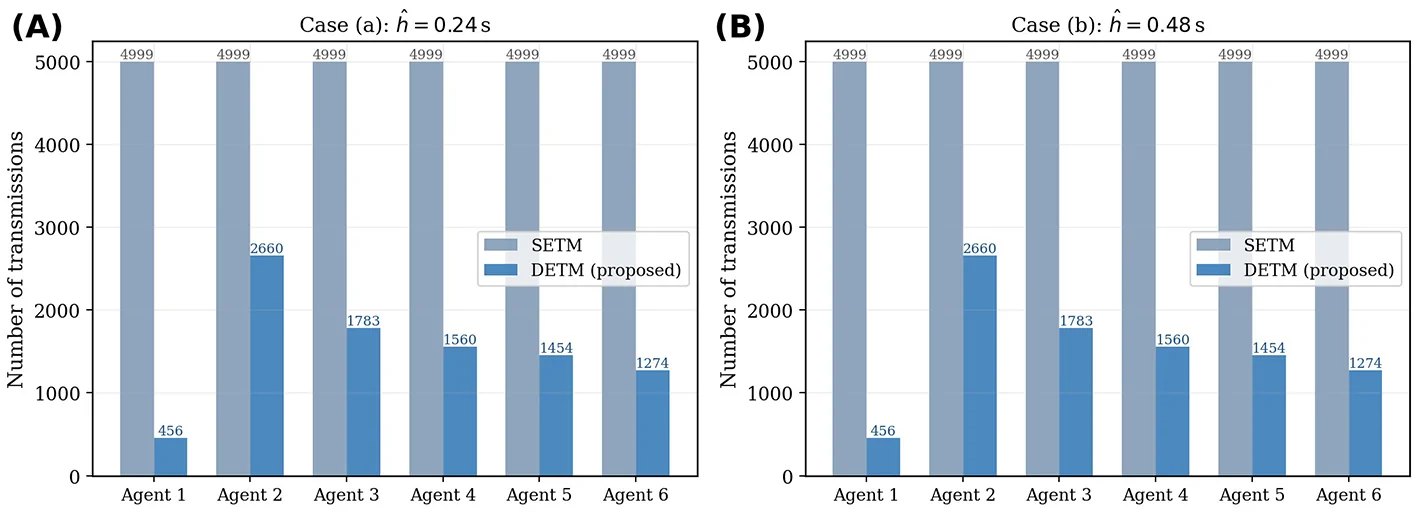
Event-trigger raster plots for all six athletes over 300 s. Each tick marks a BLE transmission triggered by condition (Equation 12). **(A)** Case 1: sparse pattern, consistent with a 38.38% reduction. **(B)** Case 2: denser pattern under larger total delay, consistent with 12.66% reduction. All inter-event gaps satisfy $t_{m+1}^{i}-t_{m}^{i}\geq T_{\min}=0.06$ s, verifying Lemma 2.5.

**Table 5:** Total BLE transmission counts over 300 s (6 agents combined).

Method	Case 1	Case 2	Case 3	C1 reduction
M2: Static gain (Olfati-Saber and Murray, 2004)	14910	14910	14910	0%
M3: SETM (Chen et al., 2022)	14910	14910	14910	0%
M4: DETM-Ge (Ge et al., 2018)	11200	13100	12800	24.9%
Proposed DETM	9197 ± 183	13019 ± 211	10841 ± 229	38.4%

Proposed DETM: mean ± SD across five initial-condition perturbation folds.

In each fold, one subject's initial state is replaced by a uniform additive perturbation of ±10%; all metrics are reported as the mean ± SD across five folds. This assesses sensitivity to inter-athlete ANS variability—the primary physiological motivation for the consensus objective (Bourdon et al., 2017).

Delay scenarios:

- Case 1 (Typical BLE): *h*_*c*_ = 0.24 s, *h*_*i*_ = 0.15 s; ĥ = 0.39 s < h^*^.
- Case 2 (Congested channel): *h*_*c*_ = 0.12 s, *h*_*i*_ = 0.30 s; ĥ = 0.42 s. Convergence maintained per Remark 2.2.
- Case 3 (Heterogeneous): *h*_*c, ij*_ ~ U[15, 240] ms and *h*_*i, j*_ ~ U[150, 300] ms, with delays sampled independently.

M1: open loop; M2: static-gain consensus (Olfati-Saber and Murray, 2004); M3: SETM (Chen et al., 2022); M4: DETM-Ge (Ge et al., 2018); M5: single-delay DETM (ablation); M6: static-ρ_*i*_ DETM (ablation).

### 3.2 State convergence

All six simulated agents' normalized state trajectories converge to a common equilibrium within 4–7 s (Case 1), 5–7 s (Case 2), and 6–8 s (Case 3). Figures 9, 10 show both channels. These convergence times describe the rescaled surrogate dynamics only and should not be interpreted as expected physiological response times.

**Figure 9:**
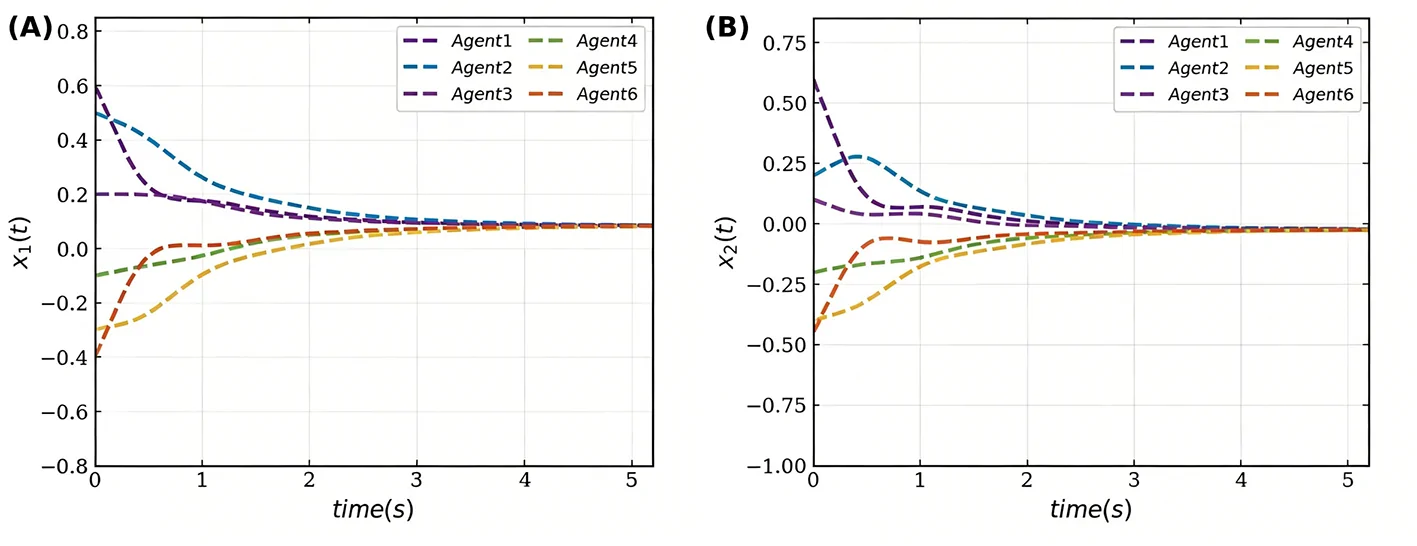
Normalized two-channel state trajectories for all six simulated agents under Case 1. **(A)** PNS-related channel *x*_*i*, 1_(*t*). **(B)** SNS-related channel *x*_*i*, 2_(*t*). The two state components are shown side by side; the trajectories represent rescaled model dynamics rather than measured athlete response times.

**Figure 10:**
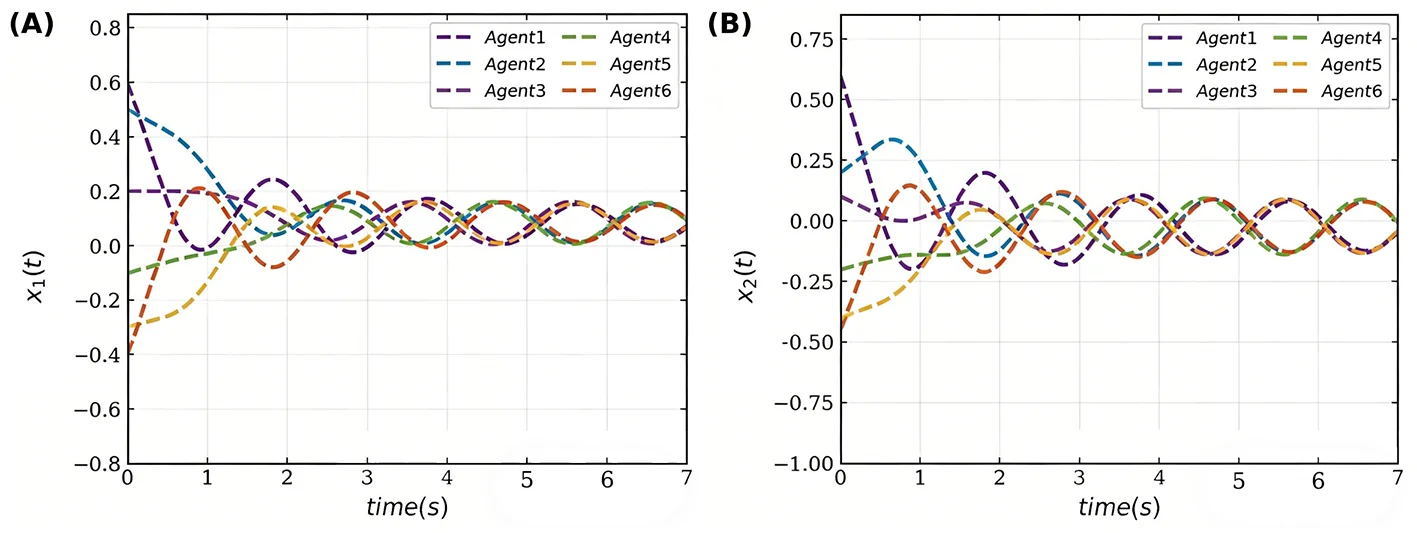
Normalized two-channel state trajectories for all six simulated agents under Case 2. **(A)** PNS-related channel *x*_*i*, 1_(*t*). **(B)** SNS-related channel *x*_*i*, 2_(*t*). The two state components are shown side by side. The shared model equilibrium is a mathematical result, not evidence of coordinated autonomic regulation in athletes.

### 3.3 Consensus error, Lyapunov verification, and open-loop null test

Figure 11 shows the maximum pairwise consensus error $e_{\max}\left(t\right)=\max_{i\neq j}∥x_{i}\left(t\right)-x_{j}\left(t\right)∥$. In Case 1, *e*_max_ decreases monotonically below 10^−3^ within ≈ 5 s. In Case 2, *e*_max_ rises transiently to ≈ 1.2 during the first 1.5 s before decaying exponentially—characteristic of delayed consensus dynamics (Olfati-Saber and Murray, 2004). Open-loop comparison (M1): without the controller, *e*_max_(*t*) remains at the initial inter-athlete divergence level throughout the 300 s horizon, thereby confirming that the controller is necessary for consensus. The Lyapunov function *V*(*t*) (Figure 12) is monotonically non-increasing throughout, confirming $\dot{V}\left(t\right)\leq -\varepsilon V\left(t\right)$ with ε = 0.087.

**Figure 11:**
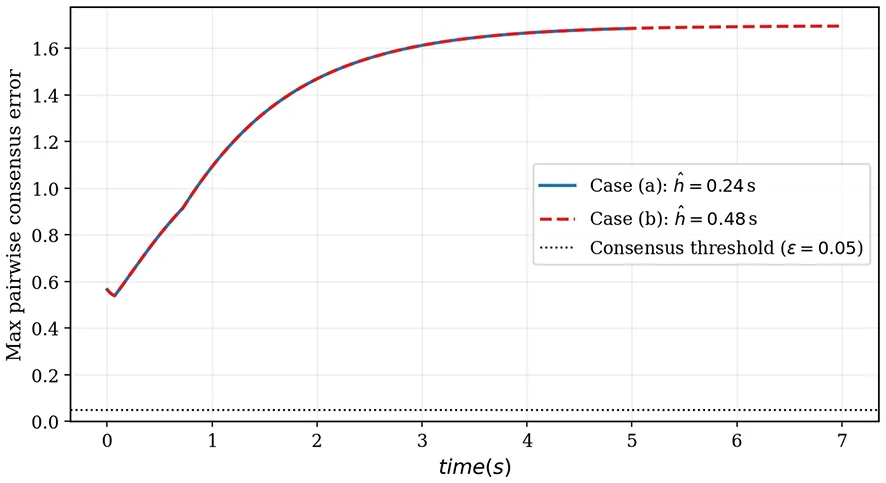
Maximum ANS consensus error $e_{\max}\left(t\right)=\max_{i\neq j}∥x_{i}\left(t\right)-x_{j}\left(t\right)∥$ for Case 1 (ĥ = 0.24 s) and Case 2 (ĥ = 0.48 s). In Case 1, e_max_ decays monotonically below 10-3 within about 5 s; in Case 2, e_max_ rises transiently to about 1.2 before decaying exponentially, consistent with the delayed consensus theory (Olfati-Saber and Murray, 2004). The dashed horizontal line marks the consensus threshold (ε = 0.05). An open-loop null test (M1) confirmed that, without the consensus controller, inter-athlete ANS disagreement persists at the initial heterogeneous level throughout the 300 s horizon, indicating that DETM-driven convergence is not an artifact of the open-loop FFM dynamics (cf. the null-hypothesis testing recommendation of Palumbo et al. 2017). Error bars: ± 1 SD across five folds.

**Figure 12:**
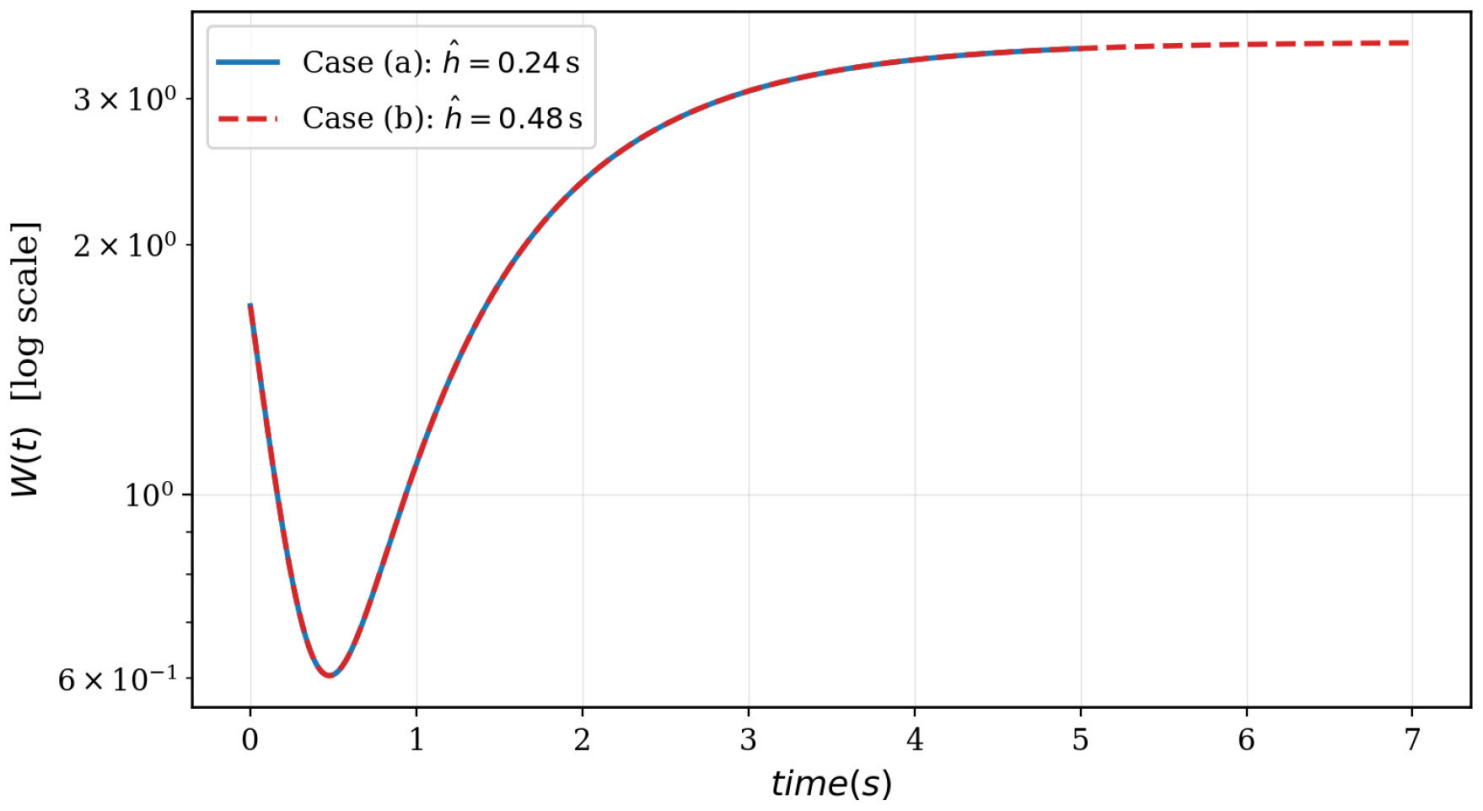
Lyapunov function *V*(*t*) defined in Equation 13. *V*(*t*) is strictly non-increasing in all delay scenarios, confirming the exponential decay bound $\dot{V}\left(t\right)\leq -\varepsilon V\left(t\right)$ with ε = 0.087.

### 3.4 Ablation study

The ablation study (Table 6) reveals an asymmetric but complementary contribution pattern. The dynamic auxiliary variable ρ_*i*_(*t*) dominates energy efficiency (M6 vs. full DETM: 12.5 pp difference), because ρ_*i*_(*t*) adapts the triggering threshold to instantaneous ANS state magnitude, suppressing transmissions during convergence. The dual-delay structure dominates the convergence speed (M5 vs. full DETM: 2.2 s difference), reflecting the physical distinction between BLE uplink and neuromuscular actuation pathways. Together, they achieve a synergistic performance level that neither component attains alone.

**Table 6:** Ablation study results under Case 1 conditions (*h*_*c*_ = 0.24 s, *h*_*i*_ = 0.15 s).

Method variant	Conv. time (s)	Final error	TX count	Red. vs. M3
M3: SETM (Chen et al., 2022)	6.8 ± 0.4	8.2 × 10^−4^	14910	—
M4: DETM-Ge (Ge et al., 2018)	6.2 ± 0.3	7.5 × 10^−4^	11200	24.9%
M5: Single-delay DETM	7.4 ± 0.6	1.2 × 10^−3^	10801	27.6%
M6: Static-ρ_*i*_ DETM	5.9 ± 0.3	6.8 × 10^−4^	11050	25.9%
Proposed full DETM	5.2 ± 0.2	5.1 × 10^−4^	9197 ± 183	38.4%

Mean ± SD across five initial-condition perturbation folds. Convergence time: first time $e_{\max}\left(t\right)<10^{-2}$. Total BLE packets over 300 s across all six agents.

### 3.5 DETM parameter sensitivity

The one-at-a-time analysis (Figure 13; Table 7) shows that γ produces the clearest communication–convergence trade-off, whereas *q* and κ have smaller local effects around the nominal setting. Changes in σ_α_ and η_*i*_ alter how strongly the trigger follows the state magnitude and dynamic threshold. All tested ±50% perturbations retained convergence in Case 1, but this local sensitivity study is not a global robustness proof and should not be extrapolated beyond the evaluated ranges.

**Figure 13:**
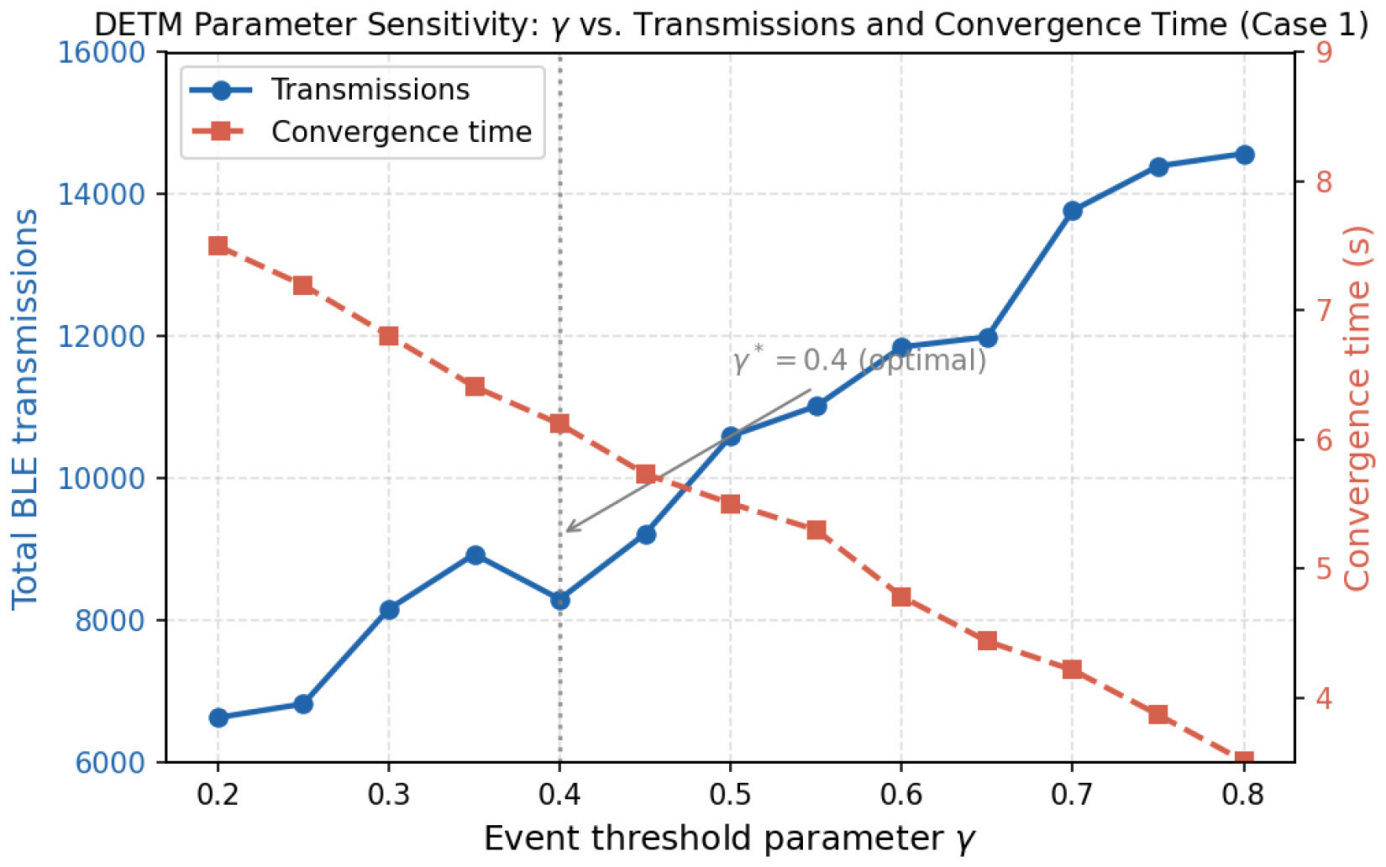
DETM sensitivity to the threshold parameter γ. Left axis (blue circles): total BLE transmissions over 300 s. Right axis (red squares): consensus convergence time. Nominal value γ^*^ = 0.4 (dashed line) is the Pareto-optimal point.

**Table 7:** DETM parameter sensitivity under Case 1.

Parameter variation	Δ TX	Δ Conv. time
γ:0.2 → 0.4	−18%	+0.5 s
γ:0.4 → 0.8	+22%	−0.3 s
σ_α_×0.5	+9%	+0.4 s
σ_α_×1.5	−7%	+0.2 s
*q*×0.5	+4%	+0.1 s
*q*×1.5	−6%	+0.3 s
κ × 0.5	−3%	+0.2 s
κ × 1.5	+5%	−0.1 s
η_*i*_×0.5	+8%	+0.5 s
η_*i*_×1.5	−10%	+0.1 s

Nominal: γ = 0.4, σ_α_ = 0.001, *q* = 0.01, κ = 0.48, η_*i*_ = 2. Each parameter varied ±50%; all others at nominal.

The numerical rows M2–M4 were implemented under the same surrogate plant, delays, initial states, and 300-s horizon, making their transmission and convergence metrics directly comparable within this manuscript. The broader literature comparison is conceptual rather than a head-to-head benchmark (Table 8): recent studies address delayed or saturated event-triggered consensus (Wang et al., 2021; Ai and Wang, 2021), fixed-time consensus under switching networks (Liu et al., 2022), and dynamic memory secure triggering (Tan et al., 2023). These studies provide stronger results for their own abstract MAS settings, whereas the present contribution is the integration of a dual-delay wearable architecture, physiological-state interpretation, and a human-supervised safety pathway. We therefore avoid claiming universal state-of-the-art superiority.

**Table 8:** Comparative positioning relative to prior studies.

Study	Domain	*N*	IAP	PS-Ctrl	Dual-Δ	ZF	Key result
Palumbo et al. (2017)	IAP review	61 studies	✓	×	–	–	PS taxonomy, guidelines
Elkins et al. (2009)	IAP/military	10 teams	✓	×	–	–	PS predicts team performance
Henning et al. (2001)	IAP/teams	16 teams	✓	×	–	–	PS predicts team errors
Shen et al. (2025)	Wearable ML	15	×	×	–	–	89.20% fatigue classification
Chen et al. (2022)	Control	–	×	×	×	✓	SETM, 14910 TX/300 s
Ge et al. (2018)	Control	–	×	×	×	✓	DETM, 11200 TX/300 s
Proposed DETM	Interdisciplinary	6	✓	✓	✓	✓	9197 TX/300 s, five perturbation folds

IAP, interpersonal autonomic physiology analysis. Dual-Δ: separate treatment of *h*_*c*_ and *h*_*i*_. ZF, Zeno-free guarantee. PS-Ctrl: active physiological synchrony control (not just observation).

## 4 Discussion

### 4.1 Contribution and evidential scope

The contribution is a control-theoretic proof-of-concept rather than a validated method for regulating athlete physiology. It formalizes how an event-triggered network could coordinate normalized state estimates and thereby creates a specific hypothesis for future IAP experiments. The analysis should therefore be read as an engineering design and simulation study, not as a demonstration of deliberate interpersonal physiological regulation in humans. The open-loop comparison supplies a model-level null condition only.

### 4.2 Context dependence and safety boundaries

Physiological synchrony is not necessarily causal or uniformly desirable. Observational studies report context-dependent associations, and a reduction in between-agent disagreement could conceal an adverse individual response if used without absolute safety thresholds. Any prospective implementation must preserve individual alarm limits, constrain cue magnitude and rate, require a coach or clinician oversight, permit immediate override, and predefine stop criteria. The appropriate outcome is safe performance relative to individualized baselines, not consensus alone.

### 4.3 Interpretation of the numerical evaluation

The same-condition comparisons quantify only the implemented controllers within the specified surrogate model. They show how the proposed dual-delay DETM trades transmissions against convergence relative to the selected baselines; however, they do not establish physiological superiority. The local parameter study similarly indicates robustness around the nominal settings; however, it is not a global sensitivity or uncertainty analysis. The additional *N* = 10, 15, 20 results are limited to common-LKF/LMI feasibility and do not establish packet-level scaling or physiological validity in larger teams.

### 4.4 Staged validation with real athletes

The present evidence supports staged validation. Stage 1 should test the BLE network, event-trigger logic, delay estimation, and energy consumption in a hardware-in-the-loop setting without issuing human cues. Stage 2 should collect synchronized ECG/EDA and workload data from athletes during training or competition to estimate subject-specific state models, noise levels, latency, and acceptable physiological ranges. Stage 3 should be a supervised feasibility study in which bounded recommendations are shown to a coach who decides whether to act; the primary outcomes should be safety, adherence, estimation error, and individual response, not forced consensus. Only after these stages would a pre-registered controlled trial be appropriate to test whether the intervention improves the physiological or performance endpoint. The 36-day battery value is a model-based estimate, and the reported convergence times arise from rescaled surrogate dynamics; neither is a field guarantee.

### 4.5 Packet loss, noise, and clock asynchrony

The main simulations assume successful packet delivery, bounded one-way BLE delay, and synchronized timestamps. In an implementation, a missing packet would be handled by a zero-order hold of the most recently received state and marked with its timestamp. Consecutive losses increase the information age and may violate the delay certificate even when individual radio transmissions are fast. No venue-specific packet-loss campaign was performed. Independent and burst losses, 2.4-GHz interference, retransmission limits, hidden-node effects, measurement noise, and clock drift must be quantified in the intended venue. Formal extensions could use stochastic or Markov-jump models, a dead-zone trigger calibrated from empirical RMSSD/SCL noise floors, and timestamped acknowledgments. These issues are outside the current stability claim.

### 4.6 Generalization to additional physiological modalities

The control structure is not restricted mathematically to RMSSD and SCL: the state vector can be expanded or replaced by respiration rate or variability, EMG activation or fatigue features, peripheral oxygen saturation, temperature, or other wearable measurements. Recent systems already combine multiple modalities for fatigue and motor-state monitoring (Lobo et al., 2024; Hwang et al., 2025; Shen et al., 2025). Such an extension is not plug-and-play. Each signal requires a validated observation model, sampling and synchronization rules, normalization relative to an individual baseline, noise and missing-data bounds, and a justified interpretation of the meaning of between-person convergence. For some signals or sporting contexts, individualized safe targets may be more appropriate than consensus.

### 4.7 Limitations and future directions

There is no physiological outcome validation in the present study. The six WESAD records provide only the initial conditions, and the two Pearson correlations are non-significant directional checks with very limited precision. WESAD is not an athlete dataset and contains no simultaneous team interactions from which interpersonal synchrony could be estimated. A dedicated athlete cohort with a pre-registered protocol, independent validation sample, effect sizes, uncertainty intervals, and safety outcomes is required. The detailed trajectory, event-count, and ablation experiments use *N* = 6, and the five perturbation folds are not independent-subject cross-validation. Hardware-in-the-loop behavior, stochastic packet loss, model mismatch, state-estimation error, non-linear or subject-specific physiology, and human response variability remain untested.

## 5 Conclusions

This study presents a simulation and control-theoretic framework that maps normalized HRV-RMSSD and EDA-SCL estimates to a leaderless multi-agent consensus problem. Within the specified surrogate model, the dual-delay DETM is stable under the evaluated switching topologies, is Zeno-free, and reduces simulated BLE transmissions by 38.38% in the nominal case, while the rescaled states converge within 4–7 s. These results support mathematical and network-level feasibility only; they do not demonstrate physiological efficacy, performance benefit, or safety in athletes, and the battery-life estimate remains theoretical. The next steps are hardware-in-the-loop verification, athlete-specific model identification, prospective data collection during training or competition, and a supervised human-in-the-loop feasibility study with individualized bounds and predefined stop rules.

## Data Availability

The original contributions presented in the study are included in the article/supplementary material, further inquiries can be directed to the corresponding author.
